# Hierarchical modelling of microbial communities

**DOI:** 10.1093/bioinformatics/btad040

**Published:** 2023-01-19

**Authors:** Manuel Glöckler, Andreas Dräger, Reihaneh Mostolizadeh

**Affiliations:** Department of Computer Science, Eberhard Karl University Tübingen, Sand 14, Tübingen 72076, Germany; Machine Learning in Science, Excellence Cluster ‘Machine Learning’, Eberhard Karl University of Tübingen, Maria-von-Linden-Str. 6, Tübingen 72076, Germany; Department of Computer Science, Eberhard Karl University Tübingen, Sand 14, Tübingen 72076, Germany; Computational Systems Biology of Infection and Antimicrobial-Resistant Pathogens, Institute for Bioinformatics and Medical Informatics (IBMI), Sand 14, Tübingen 72076, Germany; German Center for Infection Research (DZIF), Partner Site Tübingen, Wilhelmstr. 27, Tübingen 72074, Germany; Cluster of Excellence ‘Controlling Microbes to Fight Infections’, Eberhard Karl University Tübingen, Auf der Morgenstelle 28, Tübingen 72074, Germany; Department of Computer Science, Eberhard Karl University Tübingen, Sand 14, Tübingen 72076, Germany; Computational Systems Biology of Infection and Antimicrobial-Resistant Pathogens, Institute for Bioinformatics and Medical Informatics (IBMI), Sand 14, Tübingen 72076, Germany; German Center for Infection Research (DZIF), Partner Site Tübingen, Wilhelmstr. 27, Tübingen 72074, Germany; Cluster of Excellence ‘Controlling Microbes to Fight Infections’, Eberhard Karl University Tübingen, Auf der Morgenstelle 28, Tübingen 72074, Germany

## Abstract

**Summary:**

The human body harbours a plethora of microbes that play a fundamental role in the well-being of the host. Still, the contribution of many microorganisms to human health remains undiscovered. To understand the composition of their communities, the accurate genome-scale metabolic network models of participating microorganisms are integrated to construct a community that mimics the normal bacterial flora of humans. So far, tools for modelling the communities have transformed the community into various optimization problems and model compositions. Therefore, any knockout or modification of each submodel (each species) necessitates the up-to-date creation of the community to incorporate rebuildings. To solve this complexity, we refer to the context of SBML in a hierarchical model composition, wherein each species’s genome-scale metabolic model is imported as a submodel in another model. Hence, the community is a model composed of submodels defined in separate files. We combine all these files upon parsing to a so-called ‘flattened’ model, i.e., a comprehensive and valid SBML file of the entire community that COBRApy can parse for further processing. The hierarchical model facilitates the analysis of the whole community irrespective of any changes in the individual submodels.

**Availability and implementation:**

The module is freely available at https://github.com/manuelgloeckler/ncmw.

## 1 Introduction

Nasal Community Modelling Workflow (NCMW) is an open-source platform based on the Python programming language that simulates inter-species metabolism in microbial communities, focusing on the human nose. It also facilitates the development of new hypotheses about microbial interactions (Glöckler *et al.*, 2022). This package is not designed to construct genome-scale metabolic models (GEMs) of the cellular processes from scratch. The individual metabolic models of the co-occurring organisms are the building blocks of the *in silico* microbial community simulations. Therefore, any update or modification in the GEM of a given species alters the prediction of the multi-species community models (Zimmermann et al., 2021). Individual models must be accurately constructed to infer metabolic interaction networks between various organisms reliably. The NCMW takes the GEMs as input. After checking the quality of the models, the approaches constraint-based modelling, flux balance analysis (FBA) and flux variability analysis are implemented to analyse every single model further. Then, the interaction between members is analysed to determine how species share the nutrients accessible to the community. In addition, the community model created by our package is transformed into a non-linear programming, linear programming or mixed-integer linear programming problem, which conducts a dynamic FBA analysis of the community (Mahadevan *et al.*, 2002). This also creates a complex composition that is incompatible with a quick update of the available genomic sequences, causing modifications in the GEM of participation species in the community.

Consequently, these updates require an update of the community model as well. To face this challenge, we added an extensive module linked to the package NCMW, which gives the possibility of creating hierarchical models (Smith *et al.*, 2015) and is introduced in this article, along with its features. This new module coordinates with the speed at which the new GEM of participation species in the community are built or fine-tuned. Besides, it facilitates the analysis of the complex communities of multiple large species models.

## 2 Description

**Fig. 1.**
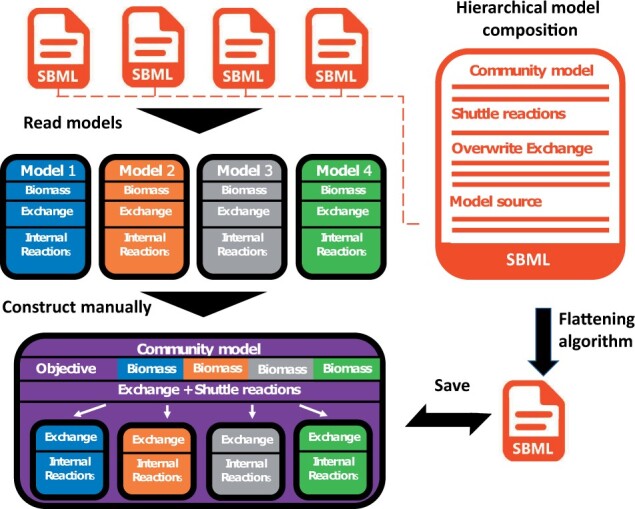
The scheme of the hierarchical model. The software combines GEMs of individual bacteria to a hierarchically structured microbiome model in SBML format and exports it to a COBRApy-compliant form for analysis and further processing

Figure 1 gives a brief overview of hierarchical models. It is often necessary to ‘manually’ construct community models from individual ones when using the NCMW or other related packages. This is not only error-prone but also requires duplicating all the information in the individual GEMs, which results in an enormous number of SBML files as the number of community members increases. Further, it decouples the community from the original files. Thus, any update within one of the underlying models of community members does not automatically propagate to the microbiome model. The Hierarchical Model Composition (‘comp’) package for SBML provides us with an alternative (Smith et al., 2015). A ‘description’ of how to combine a collection of GEMs files into a community model is constructed. The ‘description’ then only contains, e.g., the community growth objective, shuttle reaction or other constraints only relevant on a community level, as well as the Uniform Resource Identifier to the model source. As a result, the community and the individual models are linked together. Any change within the models will also affect the community.

Despite these advantages, leading optimization frameworks, such as COBRApy, do currently not support the ‘comp’ package. However, we can use the flattening algorithm, which unifies all individual models and community constraints in a single file according to the constructed ‘description’. COBRApy can effortlessly parse this file, and further analysis can be conducted: (i) CommunityModel: This gives the backbone of any community model within NCMW. (ii) BagOfReactionsModel (CommunityModel): This will create a BagOfReactionsModel from a list of models. This is equivalent to creating a model that contains the union of all reactions and metabolites within at least one model in the list. (iii) ShuttleCommunityModel (BagOfReactionsModel): This constructs a shuttle community model. Each community member exists in a separate compartment and interacts with others in an additional ‘external’ environment. So-called ‘shuttle reactions mediate the interaction between compartments and the external environment.’. (iv) HierarchicalCommunityModel (ShuttleCommunityModel): This generates a hierarchical community model. The generated model is an SBML model readable within the COBRApy. All other features defined for the community analysis on NCMW can be applied to this hierarchical community model. When the optimization is performed, it shows the optimal growth of each species within the community and the optimal growth of the entire community. Several growth media from NCMW and variable weights implemented for each species’ growth can be applied using a hierarchical community model.

## 3 Conclusion

The open-source module linked to NCMW facilitates the analysis of the complex community models built from different species. The lack of comprehensive GEMs hinders the efficiency of community analysis and optimization. As the curation of GEMs needs to be improved, and there are gaps in biological knowledge, a GEM has to be continuously updated. In this process, changes in an organism-specific model must be propagated to any community model to avoid inconsistency with the updated models. Increasing complexity may hamper this synchronization. To solve this issue, we released a new module linked to our already available package NCMW to build a hierarchical community model. The GEMs of species are imported as sub-models to create a hierarchical community model. As a result, any refinement to each model only requires rerunning the flattening algorithm to update the hierarchical community model.

Besides, this model is specified in the standard language of modelling as SBML format, which makes any further analysis of the model, such as knockout or optimization, efficient. Benchmarks of this extension using the ‘comp’ package on SBML and comparing its results with the previously created community from Glöckler et al. (2022) and Mostolizadeh et al. (2022) ensure its correctness and reliability. This new module aims to ease the analysis of microbial communities with a high-quality open-source package, thus replacing the need for culturing the species *in vitro*.

## Data Availability

The hierarchical modelling module is freely available through the open-source software NCMW and documented at https://github.com/manuelgloeckler/ncmw, including example models and data.
